# *Manihot takape* sp. nov. (Euphorbiaceae), a new tuberous subshrub from the Paraguayan Chaco

**DOI:** 10.3897/phytokeys.103.26307

**Published:** 2018-07-02

**Authors:** Juana De Egea Elsam, María del Carmen Peña-Chocarro, Fátima Mereles, Gloria Céspedes

**Affiliations:** 1 Centro para el Desarrollo de la Investigación Científica CEDIC, Fundación Moisés Bertoni para la Conservación de la Naturaleza and Laboratorios Díaz-Gill, Manduvirá 635, Asunción, Paraguay; 2 Researcher for the Programa Nacional de Incentivo a Investigadores (PRONII), Consejo Nacional de Ciencia y Tecnología (CONACYT), Paraguay; 3 The Natural History Museum, Cromwell Rd, London SW7 5BD, United Kingdom

**Keywords:** Paraguay, dry Chaco, *Manihotae*, endemism

## Abstract

*Manihot takape* De Egea & Peña-Chocarro, **sp. nov.** is described and illustrated as a new species from the Paraguayan Chaco. It was collected while carrying out fieldwork related to the study of the most important Wild Crop Relatives of the country’s flora. Morphological characteristics that differentiate this species from closely related taxa, as well as its habitat, geographical distribution and conservation status are provided.

## Introduction

*Manihot* Mill. (Euphorbiaceae) is a Neotropical genus, most likely of Mesoamerican origin, which diversified secondarily throughout South America, colonising all available types of lowland and seasonally dry environments (Deputié and Salick 2011), with the greatest centre of diversity in Brazil (Silva and Sodré 2014). The taxonomy of *Manihot* was first studied by Pohl (1827) and Pax (1910), but it was not until Rogers and Appan (1973) that the most complete taxonomic study of the genus was carried out. They recognised 98 species organised into 19 sections and distributed from Texas to Argentina. Several new species, however, have been described in recent years, mainly from Brazil and Bolivia, increasing this number to more than 120 species (Mendoza 2014, Mendoza 2016, Silva 2014, Silva 2015, Inocencio and Silva 2016, Silva et al. 2017, Lopes Martins et al. 2018). In Paraguay, the genus is represented by 15 taxa, of which 6 are endemic to the country (Rogers and Appan 1973, Peña-Chocarro and De Egea in press). The majority of them are found in the Oriental region of the country, while only four occur in the Chaco region.

During botanical expeditions to the Chaco region, while carrying out research on the most important genera of Wild Crop Relatives of the Paraguayan flora, a collection of *Manihot* was made that could not be assigned to any known species. Later, herbarium specimens with similar diagnostic characters were found and these had been misidentified as another species of *Manihot*. In this paper, we assign these specimens to a new taxon, which we describe under the name *Manihot takape* De Egea & Peña-Chocarro. The species is illustrated and its geographical distribution, ecology, phenology and conservation status are included. The new species is compared with M. anomala Pohl subsp. glabrata Chodat & Hassl. and *M. populifolia* Pax, which, in morphological terms, are the most similar taxa amongst the species found within Paraguay.

## Materials and methods

The description of this new species is based on field observations of wild populations and the examination of herbarium specimens deposited in BM, CTES, F and FCQ. Specimens of *Manihot* from Paraguay deposited in K and MA were also reviewed, but this species was not found. The holotype collection was deposited in FCQ and duplicates can be found in BM, CTES and G. The terminology used for general morphology is in compliance with Rogers and Appan (1973).

The geographic distribution map was made using ArcGIS 10.5, using georeferenced collection records. The conservation status was determined based on field observations and applying the IUCN Red List Category Criteria (IUCN 2014) and the extent of occurrence (EOO) and area of occupancy (AOO) were calculated with the Geospatial Conservation Assessment Tool (GeoCAT, http://geocat.kew.org).

## Taxonomic treatment

### Manihot takape

Taxon classificationPlantaeMalpighialesEuphorbiaceae

De Egea & Peña-Chocarro sp. nov.

urn:lsid:ipni.org:names:60476637-2

Figs 1 , 2 and 3

#### Type.

PARAGUAY. Boquerón: Neuland, Parque Valle Natural, 22°34'21"S; 60°05'31"W, 19 Feb 2018, fr., *J. De Egea, F. Mereles & S. Fernández 1793* (holotype: FCQ; isotypes: BM, CTES, G).

#### Diagnosis.

Subshrubs 0.5−0.8(−1) m tall, all parts glabrous; stems branched from base, suberect to decumbent; petiole attachment basal to occasionally narrowly peltate (less than 0.2 cm from lamina base), lamina unlobed or shallowly to deeply 3(−5)−lobed, several intermediate states found in the same plant; inflorescence a cluster of 2−6 subspicate racemes 14−33 cm long; flowers creamy-white, occasionally reddish, glabrous; pistillate flowers geminate, long pedicellate, sepals distinct, disc plicate; staminate flowers numerous, subsessile, sepals connate 1/4 their length, disc lobulate; capsules light green, unwinged, smooth when fresh, rough when dried.

**Figure 1.**
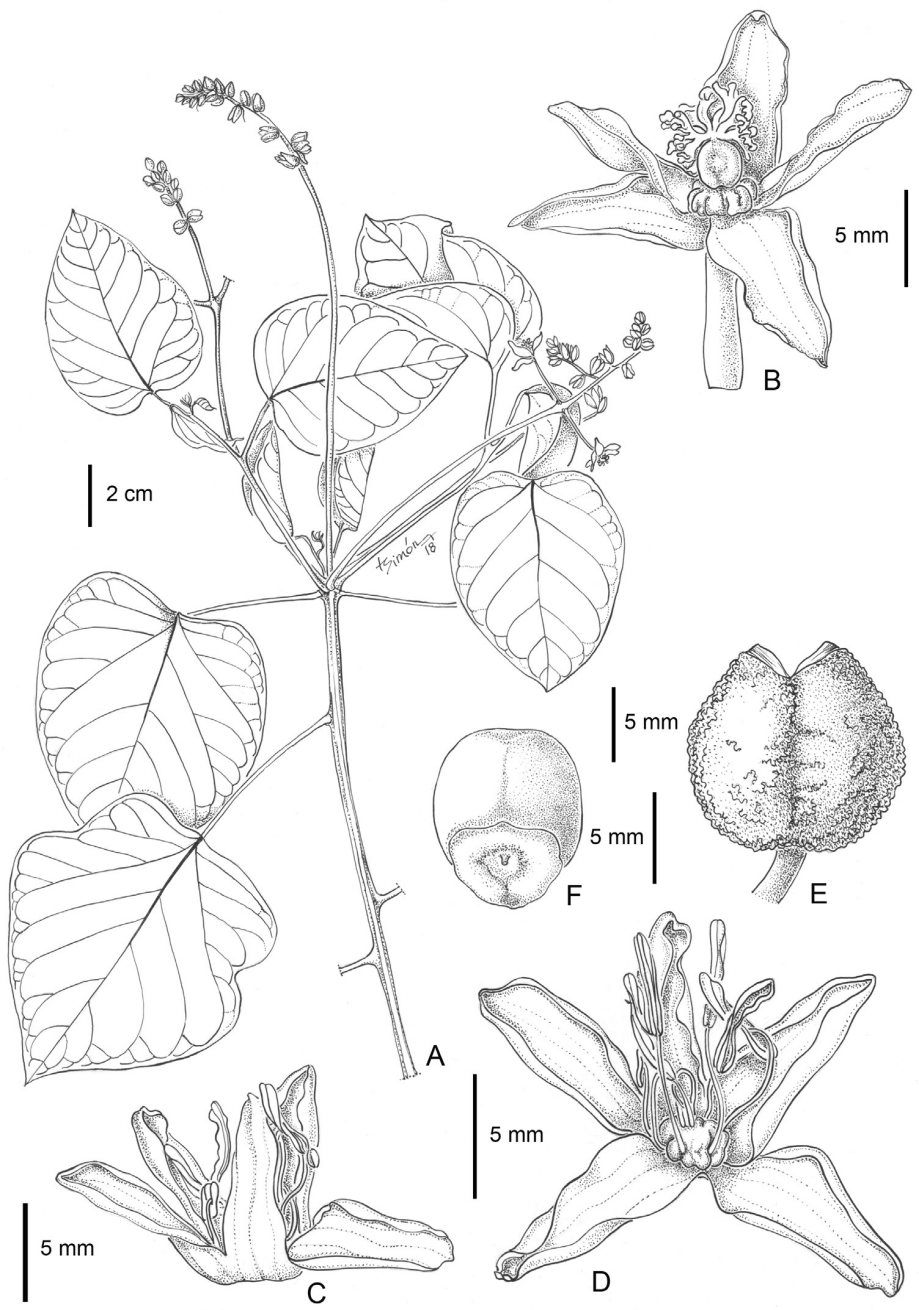
*Manihot takape*. **A** Habit (*Krapovickas & Cristóbal 44224*) **B** Pistillate flower with calyx open (*Krapovickas & Cristóbal 44224*) **C** Staminate flower (*Aquino & Quarti 470*) **D** Staminate flower with calyx split and open (*Aquino & Quarti 470*) **E** Dried capsule (*J. De Egea et al. 1793*) **F** Seed, ventral side (*J. De Egea et al. 1793*). Drawn by Laura Simón.

**Figure 2.**
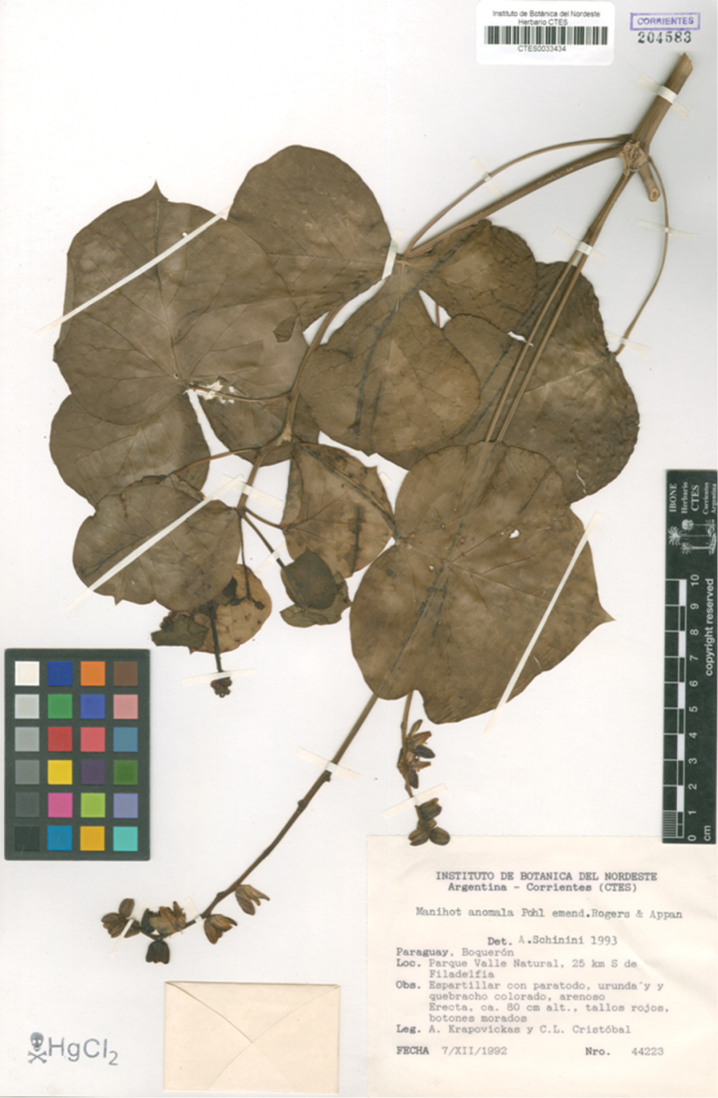
*Manihot takape*. Herbarium specimen showing different leaf forms and a particularly long inflorescence (*Krapovickas & Cristóbal 44223*). Image used with permission and provided by the Instituto de Botánica del Nordeste (CTES).

#### Description.

Subshrubs 0.5−0.8(−1) m tall. Roots slender with scattered subglobose to slightly elongated tubers, 5-10 cm diameter, ligneous outside, starchy, fibrous and creamy-white inside. Latex white. Stems branched from base, suberect to decumbent, leaning on adjacent vegetation, hollow, glabrous, green, occasionally reddish. Leaves alternate, widely spaced on stem, light green; stipules 0.2−0.3 cm long, narrowly lanceolate to filiform, margins entire, glabrous, caducous; petioles 1.5−8(−10) cm long, terete, glabrous, petiole attachment basal to occasionally narrowly peltate (less than 0.2 cm from lamina base); lamina membranaceous, smooth and glabrous on both sides, with a tuft of hairs on the adaxial side at the point of attachment of petiole, venation camptodromous; lamina unlobed to shallowly or deeply 3−lobed, sometimes with 2 additional smaller basal lobes, several intermediate states found in the same plant; sinus never reaching the lamina base, width of lamina between lamina base and sinus > 0.5 cm. Unlobed laminas 3−9(−11) × 2.5−7(−10) cm, ovate to suborbicular, base obtuse, truncate or subcordate, rarely acute, margins entire or slightly sinuate, apex acuminate; leaves mostly unlobed, especially those near the inflorescence. Lobed leaves: medial lobes 4−7 × 3−6 cm, elliptic, ovate or obovate, sometimes pandurate, apex acuminate, rarely obtuse. Inflorescence bisexual, terminal; a cluster of 2−6 racemes arising from a common base, all parts glabrous; racemes subspicate, 14−33 cm long; bracteoles and bractlets 2−3 mm long, 1 mm wide, setaceous, narrowly lanceolate, margins entire. Pistillate flowers 2, restricted to the base of the inflorescence, geminate, all parts glabrous; pedicels ca. 1−2 cm long; sepals 5, distinct, 1 cm long, creamy-white, occasionally with reddish pigmentation; disc lobed, 1 mm thick, creamy-white; ovary 3−carpellate, subglobose, styles very shortly connate, stigmas 3, profusely lobulate. Staminate flowers numerous, aggregated toward the apex of the inflorescence; pedicels 0.5 mm; buds ovoid-ellipsoid; sepals 5, connate 1/4 length, 1 cm long, creamy-white, occasionally with reddish pigmentation; disc lobed, 1 mm thick, creamy-white; stamens 8−10, filaments 3.5–5 mm long, subequal, anthers 4 mm long, oblong. Capsules 7.5−15 mm diameter, surface rough in dried specimens, to 20−23 mm diameter and smooth in fresh specimens, subglobose to slightly elongated, unwinged, apex rounded to depressed, dehiscence septicidal and loculicidal. Seeds 9−11 × 7−8 mm, 4−5 mm depth, oblong-elliptic, light glaucous greenish-grey, smooth and slightly lustrous, with few 1–2 mm dark spots towards the sides; caruncle usually prominent, light brownish-grey, opaque, extending obliquely from apex to 4 mm on the ventral side.

**Figure 3.**
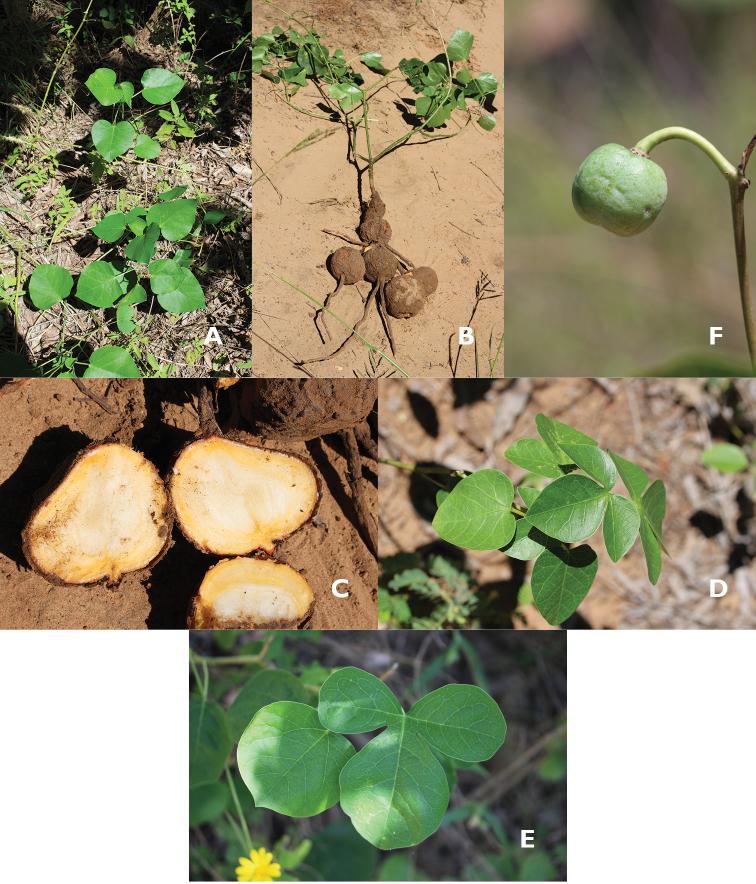
*Manihot takape (J. De Egea et al. 1793*). **A** Habit **B** Uprooted plant **C** Roots in cross-section **D–E** Leaves - note the variability in leaf forms **F** Immature fruit.

#### Distribution and ecology.

**Figure 4.**
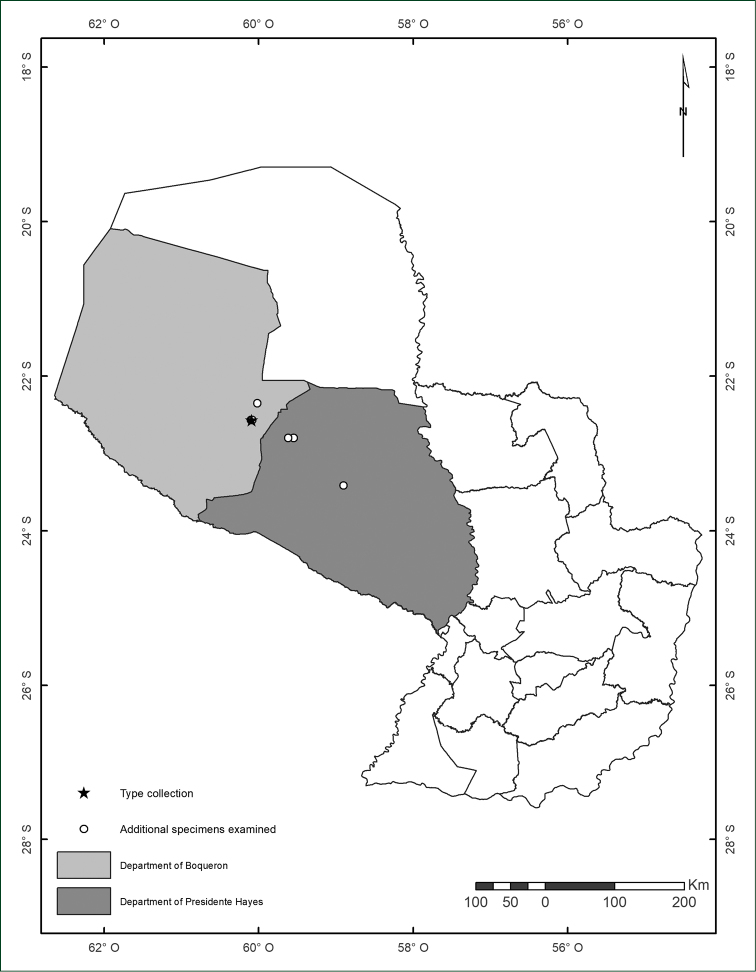
Map of Paraguay showing the known records of *Manihot takape*.

This species has been collected in dry areas of the Paraguayan Chaco, more specifically within the Departments of Boquerón and Presidente Hayes (Fig. 4). These areas are characterised by sandy and loose soils (regosols) resulting from silted palaeo-riverbeds of the Pilcomayo river delta. The species is frequent in open wooded savannahs, locally called espartillares, dominated by the grass *Elionurus muticus* (Spreng.) Kuntze (espartillo) and scattered with tree species such as *Schinopsis cornuta* Loes. (Anacardiaceae), *Astronium fraxinifolium* Schott (Anacardiaceae), *Jacaranda mimosifolia* D.Don (Bignoniaceae) and *Tabebuia aurea* Benth. & Hook.f. ex S.Moore (Bignoniaceae). Based on the data available so far, the restricted distribution of *Manihot takape* could represent an endemism of the dry Chaco. However, more surveys and collections will be needed to confirm the extension of the species distribution range.

#### Phenology.

The species has been collected with flowers and fruits from November to February and with fruits only from January to February.

#### Etymology.

The specific epithet stems from the vernacular name takape (Guarani language). This word is used for a particular habitat characterised by a wooded savannah or open woodland (Bertoni 1940). The word is also applied to small woody plants (i.e. subshrubs). This is based on the word takã (twig or branch) and the suffix ‘pe’ (short or dwarf). Both meanings fit the newly described species of *Manihot*.

#### Conservation status.

A preliminary conservation assessment with the GeoCat Tool indicates that *M. takape* can be initially considered as Endangered, with an extent of occurrence of 1,887 km^2^ and an area of occupancy of 28 km^2^ (based on an UICN default cell width of 2 km). Furthermore, with only 5 locations known to this date and considering the high deforestation and land use changes in the Paraguayan Chaco (Arévalos et al. 2018, Cardozo et al. 2013, Huang et al. 2009, Mereles and Rodas 2014, Yanosky 2013), the preliminary UICN category proposed for this species is Endangered B1ab(iii)+2ab(iii). It is worth mentioning that the type locality, known as *Parque Valle Natural* in Neuland, Boquerón, is a private reserve of approximately 0.5 km^2^ that harbours a small population of the new species. The current conservation status of the remaining four locations is unknown. Further field observations and collections are strongly recommended to achieve a more conclusive conservation assessment.

#### Additional specimens examined.

**PARAGUAY. Boquerón**: Colonia Fernheim, Colonia 22 (Neuwiese), 22°21'S, 60°01'W, 9 Nov 1987, *P. Arenas 3313* (FCQ); Neuland, Parque Valle Natural, 22°34'S; 60°06'W, 18 Jan 1993, *L. Pérez, S. Bertoni, M. Quintana, B. Benítez, G. Marín & G. Rubira 2736* (CTES); Parque Valle Natural, 25 km S de Filadelfia, 22°34'S; 60°05'W, 7 Dec 1992, *A. Krapovickas & C.L. Cristóbal 44223* (CTES); Parque Valle Natural, 25 km S de Filadelfia, 22°34'S; 60°05'W, 7 Dec 1992, *A. Krapovickas & C.L. Cristóbal 44224* (CTES, F), Parque Valle Natural, 12 km S de Filadelfia 22°34'S; 60°05'W, 28 Feb 1991, *R. Vanni, A. Radovancich & A. Schinini 2455* (CTES). **Presidente Hayes**: Colonia Menno, Paz del Chaco, 23°25'S; 58°54'W, 15 Nov 1987, *P. Arenas 3336* (FCQ); Estancia Yrendá, 22°48'S; 59°33'W, 15 Feb 1993, *L. Pérez, S. Bertoni, T. Florentín & A. Bogado 3042* (CTES); Estancia Yrendá, 22°48'S; 59°33'W, 15 Feb 1993, *L. Pérez, S. Bertoni, T. Florentín & A. Bogado 3043* (CTES); Tte. 1°Irala Fernández, próximo al centro urbano, 22°48'01"S; 59°37'05"W, 19 Feb 2012, *O. Aquino & A. Quarti 470* (FCQ).

## Discussion

*Manihot takape*, as far as known, is restricted to a particular area of the Paraguayan dry Chaco and its diagnostic characteristics and ecological associations are consistent amongst all 10 specimens examined. It stands out from other *Manihot* species of the region (taking into consideration the entire Paraguayan territory and border areas) by its predominantly unlobed leaves, the particularly long, glabrous inflorescences and its subshrubby, decumbent or “clambering” habit; the latter term, following Rogers and Appan (1973), refers to plants with stems that start erect, but may later drop over.

Due to the presence of both lobed and unlobed leaves and the basal (or nearly so) petiole attachment, the new species is morphologically most similar to Manihot anomala Pohl subsp. glabrata (Chodat & Hassl.) D.J.Rogers & Appan, from which it differs by the characters shown in Table 1. M. anomala subsp. glabrata is an erect and taller plant, frequently with a shrubby or tree-like habit up to 3 m, with mostly lobed leaves except for the ones close to the inflorescence, and inflorescences normally to 15 cm long with flowers densely pubescent to velutinous. In terms of habitat, *M. anomala* can be found in a wide range of vegetation types: subhumid and xerophytic forests, cerrado vegetation and quite frequently modified environments such as forest and trail edges, on sandy soils of variable texture and grain size. Consequently, it can be considered as a coloniser of anthropogenic environments. It has been recorded in the centre and north of the Oriental region and in all three departments of the Chaco region.

**Table 1.** Key morphological characters used to separate *Manihot takape* sp. nov., M. anomala subsp. glabrata, and *M. populifolia*.

**Character**	***M. takape* sp. nov.**	**M. anomala subsp. glabrata** ^†^	***M. populifolia*** ^†^
Habit	Subshrubs to 0.8(–1) m tall, stems suberect, decumbent or clambering, branched from base	Shrubs to 3 m tall, stems erect, generally not branched from base	Subshrubs to 0.8 m tall, stems ascending, branched from base
Indumentum	All parts glabrous, except for a tuft of hairs on the adaxial side at petiole attachment	Moderately pubescent to glabrescent. Conspicuous tuft of hairs on the adaxial side at petiole attachment	All parts glabrous, except for a tuft of hairs on the adaxial side at petiole attachment
Leaf form types	Unlobed and shallowly to deeply 3(-5) lobed	Unlobed and deeply 3(-5) lobed	Unlobed, rarely 3-lobed
Nonlobed leaves	Probably main type of leaf form, distributed in all parts of the plant, alternating with lobed leaves	Generally associated with inflorescence, close to terminal nodes	Main type of leaf form
Petiole attachment	Basal or essentially so; < 2 mm from petiole insertion to lamina base	Basal	Peltate; 2–5(–8) mm from petiole insertion to lamina base
Lamina texture	Membranaceous	Membranaceous	Membranaceous to coriaceous, with notably thickened and yellowish margin
Bracteoles and bractlets	Setaceous, 2–3 mm long, 1 mm wide	Setaceous to semifoliaceous, less than 10 mm long, 2 mm wide	Setaceous, less than 5 mm long, 1 mm wide
Inflorescence	Cluster of subspicate racemes, each 14–33 cm long	Cluster of subspicate racemes, each ca. 15 cm long	Single or cluster of 2–3 racemes, each 10(–20) cm long
Pistillate flowers	Glabrous, pedicels ca. 1–2 cm long, sepals 1 cm long	Densely pubescent to velutinous, pedicels ca. 1–2 cm long, sepals to 1–2 cm long	Glabrous, pedicels ca. 1 cm long, sepals 1 cm long
Staminate flowers	Glabrous, subsessile (pedicels 0.5 mm long), sepals 1 cm long, connate 1/4 length	Densely pubescent to velutinous, short pedicellate (pedicels 1–2 mm long), sepals 1–2 cm long, connate 1/2 length	Glabrous, pedicel length not known, sepals 1 cm long, connate1/2 length

^†^ Based on Rogers and Appan (1973) and herbarium and field observations.

Due to its subshrubby habit and predominance of unlobed leaves, *Manihot takape* can also be mistaken for *M. populifolia* Pax, from which can be easily differentiated by the petiole insertion, which is basal or essentially so in the former and peltate in the latter. In addition, the species also have different habitat preferences, with *M. populifolia* being a species known from cerrados of the Amambay and Concepción departments in the Oriental region, where it also seems to have a restricted distribution range (Zuloaga and Belgrano 2018). There is one record of *M. populifolia* from Chuquisaca, Bolivia (Jørgensen et al. 2015 onwards), but the specimen (*Pensiero & Marino 4380*, MO) could not be examined to confirm its determination. However, based on examination of the digitised image of this specimen available from TROPICOS, we believe it might be misplaced within *M. populifolia* and that it could actually be a collection of *M. takape*.

Similarities between *Manihot takape* and the two aforementioned species indicate that the new species could belong to one of their sections, *Sinuatae* or *Peltatae*, respectively, according to Rogers and Appan (1973). However, recent molecular evidence (Deputié and Salick 2011) highlights the need for a major reclassification of the genus *Manihot*. According to their results, cerrado shrubs are placed in a well supported clade that includes *M. anomala*. Undoubtedly, molecular data are needed for a complete assessment on the infrageneric placement of the newly described species.

## Supplementary Material

XML Treatment for Manihot takape

## References

[B1] Arévalos F Ortiz E Báez M Benítez C Allegretti L Duré A (2018) Monitoreo Mensual del Cambio de Uso y Cobertura de la Tierra, Incendios y Variación de la Cubierta de Aguas en el Gran Chaco Americano; Enero 2018. Guyra Paraguay. http://guyra.org.py/informe-deforestacion

[B2] Bertoni MS (1940) Diccionario Botánico Latino-Guaraní y Guaraní-Latino con un glosario de vocablos y elementos de la nomenclatura botánica. Editorial Guaraní, Asunción, 99−100.

[B3] Cardozo R Palacios F Rodas O Yanosky A (2013) Cambio en la cobertura de la tierra del Gran Chaco Americano en el año 2012. Paraquaria Natural 1(2): 43−49. http://guyra.org.py/paraquaria-2013/

[B4] Deputié A Salick J (2011) Evolutionary biogeography of *Manihot* (Euphorbiaceae), a rapidly radiating Neotropical genus restricted to dry environments. Journal of Biogeography 38(6): 1033–1043. https://doi.org/10.1111/j.1365-2699.2011.02474.x

[B5] Huang G Kim S Song K Townshend JRG Davis P Altstatt A Rodas O Yanosky A Clay R Tucker CJ Musinsky J (2009) Assessment of Paraguay’s forest cover change using Landsat observations. Global and Planetary Change 67(1–2): 1–12. https://doi.org/10.1016/j.gloplacha.2008.12.009

[B6] Inocencio LS Silva MJ (2016) A vine-like species of *Manihot* (Euphorbiaceae) from the state of Mato Grosso, Brazil. Systematic Botany 41(4): 983–988. https://doi.org/10.1600/036364416X694107

[B7] IUCN (2014) Guidelines for using the IUCN red list categories and criteria (Ver. 11). IUCN, Gland, Switzerland and Cambridge. http://cmsdocs.s3.amazonaws.com/RedListGuidelines.pdf

[B8] Jørgensen PM Nee MN Beck SG (2015 onwards) Catálogo de las plantas vasculares de Bolivia. Missouri Botanical Garden. http://www.tropicos.org/projectwebportal.aspx?pagename=Home&projectid=13 [accessed: 29.04.2018]

[B9] Lopes Martins ML Lemos de Carvalho PC da Silva Ledo CA Amorim AM (2018) *Manihot alternifolia* and *M. elongata* spp. nov. (Euphorbiaceae) and the rediscovery of *M. quinquefolia* in Caatinga (semiarid) vegetation in Brazil. Nordic Journal of Botany 36(3): 1–8. https://doi.org/10.1111/njb.01615

[B10] Mendoza FJM (2014) *Manihot* (Euphorbiaceae) en Bolivia: Parte I: Tres especies nuevas y un nuevo registro. Brittonia 66(2): 107–117. https://doi.org/10.1007/s12228-013-9303-3

[B11] Mendoza FJM (2016) Taxonomic novelties in *Manihot* (Euphorbiaceae) from Bolivia and adjacents areas. Revista de la Sociedad Boliviana de Botánica 9(1): 7–16.

[B12] Mereles F Rodas O (2014) Assessment of rates of deforestation classes in the Paraguayan Chaco (Great South American Chaco) with comments on the vulnerability of forest fragments to climate change. Climatic Change 127(1): 55–71. https://doi.org/10.1007/s10584-014-1256-3

[B13] Pax F (1910) *Manihot* Adans – Das Pflanzenreich IV. 147 II, Heft 44, ed. HGA Engler. Wilhelm Engelmann, Leipzig, 21−111.

[B14] Peña-Chocarro MC De Egea J (in press) Checklist of endemic vascular plants of Paraguay. Phytotaxa.

[B15] Pohl J (1827) Plantarum Brasiliae Icones and Descriptions. 1: 17–56.

[B16] Rogers DJ Appan SG (1973) *Manihot* and *Manihotoides* (Euphorbiaceae). A computer assisted study. Flora Neotropica (Monograph no. 13). Hafner Press, New York.

[B17] Silva MJ (2014) *Manihot veadeirensis* (Euphorbiaceae s. s.): A new species from the Brazilian Cerrado. Systematic Botany 39(4): 1161–1165. https://doi.org/10.1600/036364414X682625

[B18] Silva MJ (2015) *Manihot apanii* (Euphorbiaceae s.s.) a new species from Brazil, and a key to the species with unlobed or very shortly lobed leaves. Systematic Botany 40(1): 168–173. https://doi.org/10.1600/036364415X686477

[B19] Silva MJ Inocencio LS Sodré RC Alonso AA (2017) Morphological and anatomical evidence support a new wild cassava: *Manihot fallax* (Crotonoideae, Euphorbiaceae), from Mato Grosso, Brazil. PhytoKeys 91: 139–156. https://doi.org/10.3897/phytokeys.91.21465 10.3897/phytokeys.91.21465 PMC5769679 29362549

[B20] Silva MJ Sodré RC (2014) A dwarf species of *Manihot* Mill. (Euphorbiaceae s. s.) from the highlands of Goiás, Brazil. Systematic Botany 39(1): 222–226. https://doi.org/10.1600/036364414X678134

[B21] Yanosky A (2013) The challenge of conserving a natural Chaco habitat. Paraquaria Natural 1(1): 32−34. http://guyra.org.py/paraquaria-2013/

[B22] Zuloaga FO Belgrano JM (2018) Catálogo de plantas vasculares del Cono Sur. http://www.darwin.edu.ar/proyectos/floraargentina/fa.htm [accessed: 29.04.2018]

